# The impact of impulse control disorders on cognitive decline in *de novo* Parkinson’s disease: a study based on structural MRI

**DOI:** 10.3389/fneur.2025.1565046

**Published:** 2025-04-25

**Authors:** Ruohong Xu, Beisheng Yang, Juanling Li, Xiaofan Wei, Limin Zhang, Jiaqi Tian, Wei Zhang

**Affiliations:** Department of Radiology, The Second Affiliated Hospital of Chongqing Medical University, Chongqing, China

**Keywords:** Parkinson’s disease, impulse control disorders, mild cognitive impairment, cortical thickness, survival analysis

## Abstract

**Background:**

Impulse control disorders (ICDs) are common neuropsychiatric symptoms (NPS), which are prevalent among patients with Parkinson’s disease (PD). Current research has not clarified the impact of ICDs on cognitive function nor provided sufficient objective evidence. This study aims to explore the effects of ICDs on cognitive functions in PD patients, and further investigate associated cerebral structural changes.

**Methods:**

Two hundred PD patients with normal cognition (PDNC) and 69 healthy controls were included from the Parkinson’s Progression Markers Initiative (PPMI), among these PDNC, 81 patients with “pure” ICDs (p-ICDs), 69 ICDs combined with other NPS (c-ICDs), and 50 patients without NPS. The cognitive status of each PD patient was obtained every year in four-year follow-up. The difference in conversion rates was obtained by chi-square test. Survival analysis was used to explore the conversion time difference among these groups. Further analysis was conducted on the potential structural difference. Finally, the correlation between significant brain structural changes and neuropsychological assessments were evaluated.

**Results:**

The survival analysis suggested that the conversion time of p-ICDs from normal cognition to MCI was significantly delayed compared to NPS-negative, with no significant difference relative to the c-ICDs. There is no significant difference in conversion rates among them. Morphological analysis revealed that compared to the NPS-negative group, the p-ICDs and c-ICDs groups exhibited thickness changes in certain regions (Bonferroni-corrected, *p* < 0.05).

**Conclusion:**

Our findings suggest that ICDs might exert a protective effect against cognitive decline, potentially delay the occurrence of MCI in PDNC, which could be associated with alterations in cortical thickness.

## 1 Introduction

Cognitive deficits are commonly observed in Parkinson’s disease (PD) (1). Approximately 30–40% of individuals may exhibit mild cognitive impairment (MCI) early in the disease course (2, 3), which might emerge before or at the time of PD diagnosed. Previous studies have shown that MCI is a risk factor for Parkinson’s disease dementia (PDD) and an established stage of the condition (4, 5). Compared to PD patients with normal cognition (PDNC), PDMCI have a higher risk of developing to PDD (6, 7). Almost 60–80% of PDMCI eventually develop to PDD (8), which would significantly impact their quality of life and tend to deteriorate as the disease progress (9).

Besides cognitive impairment, the neuropsychiatric symptoms (NPS), such as depression, anxiety, apathy, psychosis and impulse control disorders (ICDs), are also common non-motor symptoms in PD patients (10). These clinical signs and symptoms may present either independently or simultaneously (11). Among these NPS, ICDs, which include compulsive, repetitive, and excessive behaviors such as gambling, sexual activity, shopping, and eating (12), have a relatively high incidence and are often accompanied by other NPS (13–15). For decades, the relationship between NPS and cognitive impairment in Parkinson’s disease has sparked considerable interest among researchers. Some studies found that NPS, such as depression and anxiety, apathy, are associated with cognitive impairment in Parkinson’s disease and serve as predictors for cognitive decline (16–20). While, other researchers indicated that PD with the single and coexistence of NPS exhibit relatively lower cognitive decline within a certain period (21–23). Due to the inconsistencies of these findings and the inherent limitations of neuropsychological assessments, this issue still remains controversial.

Structural magnetic resonance imaging provides an objective and non-invasive method to accurately measure changes in brain structure, providing biomarkers for monitoring cognitive decline, an advantage over conventional clinical assessment (24). To date, there have been few morphometric analyses on the impact of ICDs on cognitive function in PD. Biundo et al. found that cognitive function was relatively preserved in PD with ICD, but voxel-based morphometry analysis revealed no significant differences between PD with and without ICDs (25). Therefore, the impact of ICDs combined with other NPS on cognitive changes in PD and the relevant imaging evidence needs to be further explored.

In this study, we aim to investigate that (1) whether single ICDs and ICDs combined with other NPS have an impact on the progression of cognitive impairment; (2) The structural differences between single ICDs and ICDs combined with other NPS; (3) the correlation between cortical changes and cognitive assessments.

## 2 Materials and methods

### 2.1 Participants

The data were acquired from PPMI^1^ (26). The PPMI study protocols were approved by local ethics committees, which involved all 33 clinical research institutions. Written informed consent was obtained prior to their inclusion in the study. Methods in this study were in accordance with relevant guidelines and regulations. The inclusion criteria of PD patients were as follows: (I) the patients must have at least 2 of the following: resting tremor, bradykinesia, rigidity, and either asymmetric resting tremor or asymmetric bradykinesia; (II) At baseline, the patients remained untreated; during the follow-up period, they were required to strictly adhere to the prescribed dosage and medication protocols; (III) the patients have T1-weighted images without distortion or head motion artifacts. The exclusion criteria were as follows: (I) Cognitive decline caused by other diseases (such as brain tumors, cerebral hemorrhages, etc.); (II) the patient has taken PD medication recently; (III) MRI images are missing or of poor quality; (IV) The subjects were diagnosed with PDD.

The cognitive assessments were evaluated at baseline and with 12 months interval in four-year follow-up. Based on the cognitive assessments, the cognitive status of all participants were obtained in four-year follow-up. Patients were classified as PDMCI if they met one of the following criteria: (I) cognitive impairment evidenced by the Montreal Cognitive Assessment (MoCA) (27, 28), MOCA<26 as a cut-off, or (II) scores below 1.5 standard deviations from the age/education normative mean on at least two neuropsychological tests, including the Hopkins Verbal Learning Test (HVLT), Judgment of Line Orientation (JLO), Letter Number Sequencing (LNS), Semantic Fluency Test (SFT), and Symbol Digit Modality Test (SDMT) (29).

Neuropsychiatric symptoms were assessed at baseline, Depression was evaluated with the 15-item Geriatric Depression Scale (GDS-15), and a scores ≥5 indicated clinically significant depression (30). Anxiety was evaluated with the State–Trait Anxiety Inventory (STAI), and a score ≥40 on any subscale was considered as clinically significant anxiety (31). ICDs and related behaviors were assessed with the short form of the Questionnaire for ICDs in PD (32). Psychosis and apathy were separately assessed through the MDS-Psychiatric Rating Scale 17, part I, focusing on the hallucination/psychosis and apathy items (33). The presence of any level 1 or higher symptoms of psychosis or apathy was considered indicative of these conditions.

Following a series of screenings, 200 PD patients with normal cognition (PDNC) were diagnosed with ICDs. Taking into account the mediating effects of other neuropsychiatric symptoms, among these PDNC, 81 participants which had ICDs only were diagnosed as “pure” ICDs (p-ICDs), while 69 PD-ICDs with other NPS such as depression, anxiety, psychosis, and apathy were diagnosed as PD-ICDs combined other NPS (c-ICDs). Fifty PD patients without NPS were classified as NPS-negative. The study also included 69 healthy controls (HC).

### 2.2 Image acquisition

The magnetic resonance imaging parameters were standardized across all scanners with a slice thickness of 1 mm and a matrix size of 256 × 256. For GE MEDICAL SYSTEMS scanners, the Signa HDxt model used a field of view (FOV) of 250 mm, echo time (TE) of 3.6 ms, inversion time (TI) of 450.0 ms, repetition time (TR) of 9.1 ms, and a flip angle of 13.0°; the SIGNA EXCITE model featured a FOV of 230 mm, TE of 4.0 ms, TI of 0.0 ms (no inversion recovery), TR of 8.3 ms, and a flip angle of 15.0°; while the DISCOVERY MR750 employed a FOV of 260 mm, TE of 4.2 ms, TI of 450.0 ms, TR of 8.2 ms, and a flip angle of 13.0°. Siemens scanners included the Espree (FOV = 250 mm, TE = 3.2 ms, TI = 1,100.0 ms, TR = 1,970.0 ms, flip angle = 15.0°), Verio (FOV = 240 mm, TE = 3.0 ms, TI = 900.0 ms, TR = 2,300.0 ms, flip angle = 9.0°), and TrioTim (FOV = 250 mm, TE = 3.0 ms, TI = 900.0 ms, TR = 2,300.0 ms, flip angle = 9.0°). Philips Medical Systems scanners comprised the Achieva (FOV = 220 mm, TE = 3.2 ms, TI = 0.0 ms, TR = 7.0 ms, flip angle = 8.0°) and Intera (FOV = 250 mm, TE = 4.0 ms, TI = 0.0 ms, TR = 8.5 ms, flip angle = 8.0°), with TI = 0.0 ms indicating no inversion recovery (Table 1). And the number of participants scanned across eight different manufacturers are provided as follows (Table 2).

**Table 1:** MRI image acquisition protocols of this study.

Manufacturer	Slice thickness	Matrix size	Fov	TE	TI	TR	Flip angle
GE MEDICAL SYSTEMS, Signa HDxt	1 mm	256 × 256	250 mm	3.6 ms	450.0 ms	9.1 ms	13.0 degree
GE MEDICAL SYSTEMS, SIGNA EXCITE	1 mm	256 × 256	230 mm	4.0 ms	0.0 ms	8.3 ms	15.0 degree
GE MEDICAL SYSTEMS, DISCOVERY MR750	1 mm	256 × 256	260 mm	4.2 ms	450.0 ms	8.2 ms	13.0 degree
SIEMENS, Espree	1 mm	256 × 256	250 mm	3.2 ms	1,100.0 ms	1,970.0 ms	15.0 degree
SIEMENS, Verio	1 mm	256 × 256	240 mm	3.0 ms	900.0 ms	2,300.0 ms	9.0 degree
SIEMENS, TrioTim	1 mm	256 × 256	250 mm	3.0 ms	900.0 ms	2,300.0 ms	9.0 degree
Philips Medical Systems, Achieva	1 mm	256 × 256	220 mm	3.2 ms	0.0 ms	7.0 ms	8.0 degree
Philips Medical Systems, Intera	1 mm	256 × 256	250 mm	4.0 ms	0.0 ms	8.5 ms	8.0 degree

**Table 2:** The number of participants scanned across eight different manufacturers.

Manufacturer	p-ICDs (*n* = 81)	c-ICDs (*n* = 69)	NPS-negative (*n* = 50)	HC (*n* = 69)
GE MEDICAL SYSTEMS, Signa HDxt	12	9	8	10
GE MEDICAL SYSTEMS, SIGNA EXCITE	9	8	5	8
GE MEDICAL SYSTEMS, DISCOVERY MR750	9	7	6	8
SIEMENS, Espree	9	9	5	8
SIEMENS, Verio	7	6	5	7
SIEMENS, TrioTim	14	12	8	10
Philips Medical Systems, Achieva	9	7	5	8
Philips Medical Systems, Intera	12	11	8	10

### 2.3 Image processing

Cortical thickness was assessed by *FreeSurfer*,^2^ which computed the average distance between the gray/white matter boundary and the pial surface at each vertex on the cortical surface. Employing well-established FreeSurfer processing pipeline (34), a robust, unbiased subject-specific template was generated through inverse consistent registration across all available MRI scans for each participant (35, 36). Several processing steps, such as skull stripping, Talairach transformation, atlas registration, and the creation of spherical surface maps and parcellations, were executed based on the subject-specific templates. The cerebral cortex was parcellated into 68 distinct anatomical regions, within which the average thickness was determined. Prior to further analysis, each individual brain map underwent visual inspection to ensure proper registration.

### 2.4 Statistical analysis

Statistical analysis was performed using SPSS software 27.0. For quantitative data that followed a normal distribution, inter-group comparisons were conducted using one-way ANOVA. Non-parametric tests were conducted using the Kruskal-Wallis test. For multiple comparisons, the Bonferroni correction was applied to adjust the probability of Type I errors. Categorical data were analyzed using the Chi-square test, with a significance level of *α* = 0.05. Subsequently, with progression to MCI as the status variable and follow-up time as the time variable, differences in conversion rates among the p-ICDs, c-ICDs, NPS-negative and HC were investigated using the Kaplan–Meier survival analysis. The power analysis was performed through univariate ANOVA methodology to systematically validate the reliability of cognitive function assessments. Differences in cortical thickness among groups were further compared using one-way ANOVA then. Based on this, Pearson analysis was used to explore the correlation between cortical thickness and neuropsychological tests, with a significance level of *α* = 0.05.

## 3 Results

### 3.1 Demographics and cognitive analysis

At baseline, no significant differences were observed in age, sex, years of education, and H&Y stages among the p-ICDs group, c-ICDs group, NPS-negative group, and the control group (*p* > 0.05).

The HVLT, JLO, LNS, SFT and SDMT showed no significant differences at baseline across the p-ICDs group, c-ICDs group, NPS-negative group, and the control group (*p* > 0.05) (Table 3).

**Table 3:** Cognitive data and basic characteristics of Parkinson’s disease patients with p-ICDs, c-ICDs, NPS-negative and healthy control participants.

Clinical data	p-ICDs (*n* = 81)	c-ICDs (*n* = 69)	NPS-negative (*n* = 50)	HC (*n* = 69)	*p* value
Age (year)	61.67 ± 9.56	61.88 ± 9.65	63.13 ± 10.32	61.13 ± 11.82	0.758^a^
Sex (female/male)	29/52	30/39	19/31	26/43	0.803^b^
Education duration	15.97 ± 2.92	16.30 ± 3.36	16.20 ± 3.28	16.20 ± 2.8	0.907^a^
H&Y	1.55 ± 0.56	1.72 ± 0.54	1.68 ± 066		0.769^d^
UPDRS-III	20.08 ± 10.73	22.31 ± 9.35	23.51 ± 9.56		0.933^d^
MoCA	27.80 ± 1.92	27.32 ± 1.81	27.96 ± 2.22	28.45 ± 1.17	0.088^a^
JLO	12.52 ± 2.52	11.84 ± 3.01	11.44 ± 2.43	12.74 ± 2.52	0.522^a^
LNS	53.00 ± 9.21	51.68 ± 10.26	52.04 ± 11.07	53.52 ± 9.88	0.820^c^
HVLT	49.80 ± 8.81	49.33 ± 9.92	49.60 ± 9.84	49.61 ± 9.72	0.829^c^
SDMT	46.28 ± 8.72	48.32 ± 8.60	46.28 ± 7.58	46.48 ± 9.75	0.720^c^
SFT	53.00 ± 9.21	51.68 ± 10.26	52.04 ± 11.07	53.52 ± 9.88	0.673^c^

Quantitative data are presented as mean ± standard deviation if normally distributed, or median ± interquartile range if not. *p* < 0.05 indicates statistical significance. a = Kruskal-Wallis test, b = Chi-square test, c = One-way ANOVA, d = Mann–Whitney U test.

### 3.2 Survival analysis and conversion rate analysis

Among 81 p-ICDs, 69 c-ICDs, and 50 NPS-negative, the conversion rates to MCI were 20, 28, and 38%, respectively. And there was no significant difference in the conversion rates among these groups (*p* = 0.073) (Supplementary Table S1).

**Figure 1:**
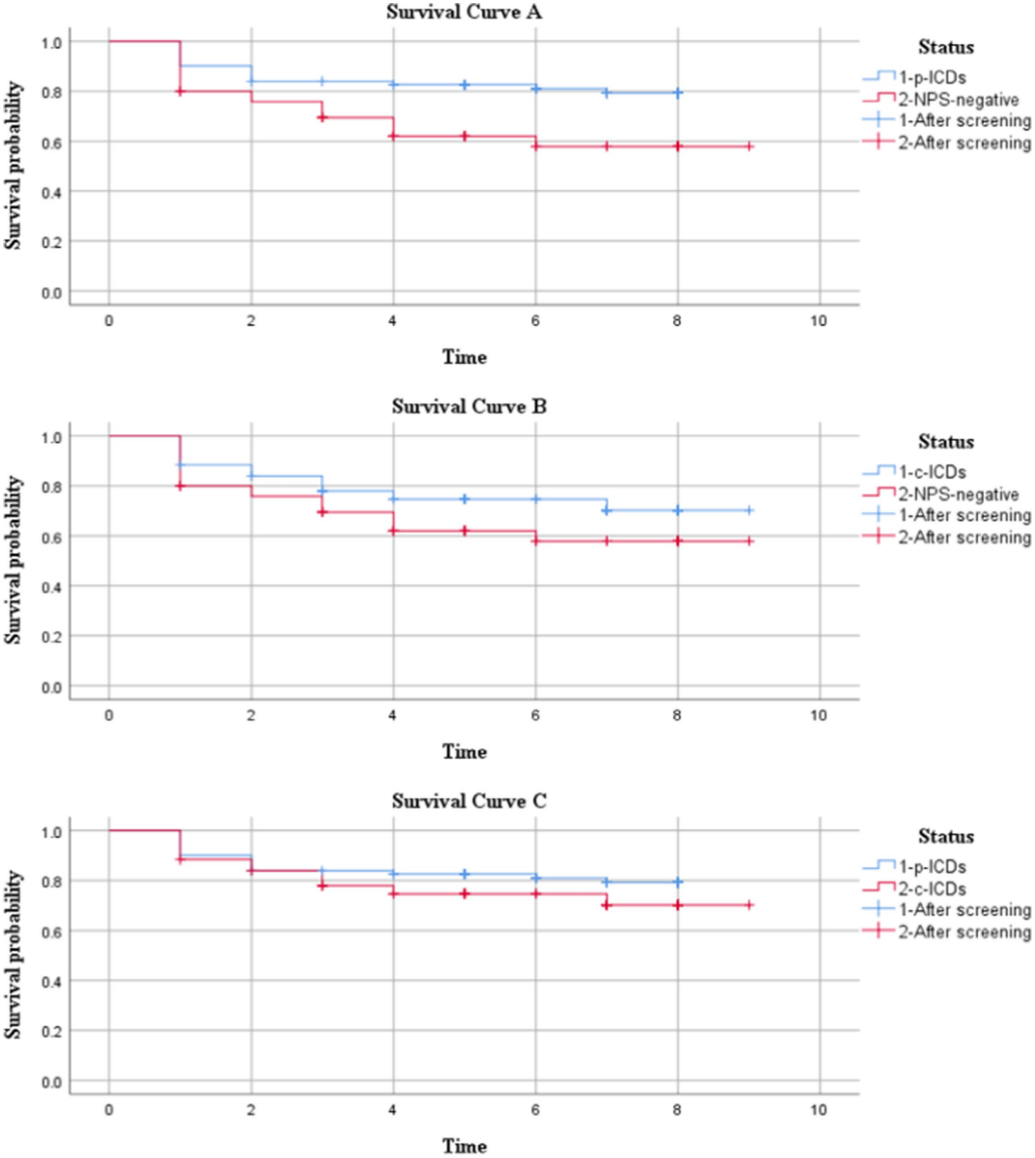
Survival analysis of Parkinson’s disease patients with p-ICDs and NPS-negative, c-ICDs and NPS-negative, p-ICDs and c-ICDs. **(A)** p-ICDs VS NPS-negative, **(B)** c-ICDs VS NPS-negative, **(C)** p-ICDs VS c-ICDs.

The Kaplan–Meier analysis revealed that the time in the p-ICDs group to progress from normal cognition to MCI was significantly longer than that of the NPS-negative group. (*p* = 0.011) (Figure 1A). There were no significant difference in conversion rates between the c-ICDs group and NPS-negative group (*p* = 0.152) (Figure 1B), p-ICDs group and c-ICDs group (*p* = 0.250) (Figure 1C).

### 3.3 Analysis of differential brain regions

Compared to the HC, both p-ICDs group and c-ICDs group exhibited thinning in the bilateral caudal cingulate cortex (cACC), bilateral frontal pole and bilateral isthmus cingulate cortex (ICC). Similarly, both c-ICDs group and NPS-negative group exhibited thinning in the right rostral middle frontal gyrus (rMFG) in contrast to the HC. Additionally, the p-ICDs group showed thinning in the right parahippocampal gyrus (PHG) and right rostral anterior cingulate cortex (rACC) in comparison to HC (Bonferroni-corrected, *p* < 0.05, Table 4).

**Table 4:** Differences in cortical thickness among patients with p-ICDs, c-ICDs, NPS-negative and healthy control participants.

Brain area	p-ICDs (*n* = 81)	c-ICDs (*n* = 69)	NPS-negative (*n* = 50)	HC (*n* = 69)	*p* value
Left cACC	2.37 ± 0.23	2.37 ± 0.28	2.38 ± 0.25	2.51 ± 0.31	0.002^de^
Left iCC	2.20 ± 0.11	2.16 ± 0.12	2.28 ± 0.17	2.33 ± 0.27	0.000^abcde^
Left paORB	2.52 ± 0.23	2.57 ± 0.21	2.58 ± 0.17	2.71 ± 0.29	0.003^ef^
Left frontal pole	2.64 ± 0.22	2.65 ± 0.23	2.66 ± 0.24	2.74 ± 0.25	0.018^de^
Right cACC	2.24 ± 0.20	2.25 ± 0.22	2.28 ± 0.21	2.37 ± 0.31	0.003^de^
Right iCC	2.23 ± 0.15	2.18 ± 0.16	2.27 ± 0.18	2.35 ± 0.29	0.000^de^
Right PHG	2.54 ± 0.20	2.61 ± 0.24	2.65 ± 0.26	2.70 ± 0.29	0.000^bd^
Right rACC	2.58 ± 0.23	2.61 ± 0.25	2.63 ± 0.28	2.71 ± 0.35	0.031^d^
Right rMFG	2.25 ± 0.16	2.26 ± 0.12	2.26 ± 0.13	2.38 ± 0.17	0.002^ef^
Right frontal pole	2.62 ± 0.22	2.63 ± 0.25	2.62 ± 0.27	2.51 ± 0.23	0.008^de^

a. p-ICDs VS c-ICDs b. p-ICDs VS NPS-negative c. c-NPS VS NPS-negative d. p-ICDs VS HC e. c-ICDs VS HC f. NPS-negative VS HC.

cACC, caudal anterior cingulate cortex; rACC, rostral anterior cingulate cortex; paORB, pars orbitalis; PHG, parahippocampal gyrus; iCC, isthmus cingulate cortex; rMFG, rostral middle frontal gyrus.

**Figure 2:**
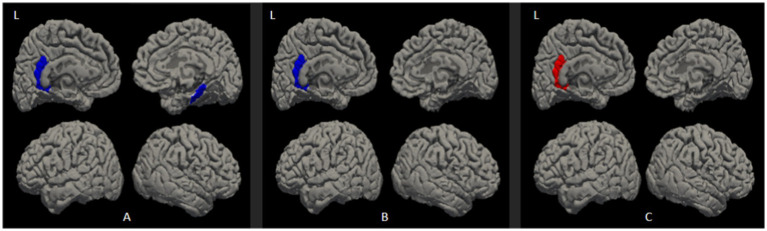
Differential cortical thickness map between group comparison. **(A)** p-ICDs VS NPS-negative, **(B)** c-ICDs VS NPS-negative, **(C)** p-ICDs VS c-ICDs. Blue represents areas of cortical thinning, while red represents areas of cortical thickening.

Compared to the NPS-negative group, both the p-ICDs group and the c-ICDs group showed significant thinning in the left ICC, and the p-ICDs group exhibited thinning in the right PHG (Bonferroni-corrected, *p* < 0.05, Figure 2). The p-ICDs group exhibited thickening in the left ICC compared to the c-ICDs group (Bonferroni-corrected, *p* < 0.05, Table 4; Figure 2).

The *post hoc* power analysis showed that the Cohen *d* (Eta squared) between the three groups (p-ICDs, c-ICDs, NPS negative) was 0.72, reaching a moderate effect (Supplementary Figure S2).

### 3.4 Correlation analysis

The correlation analysis was conducted between the significantly altered cortical regions and clinical neuropsychiatric tests (Supplementary Figure S1). The left cACC was negatively correlated with the Judgment of Line Orientation in the p-ICDs group (*r* = −0.220, *p* = 0.049). The right PHG showed a positive correlation with the Hopkins Verbal Learning Test in p-ICDs group (*r* = 0.376, *p* = 0.001) and HC group (*r* = 0.239, *p* = 0.048), but showed a negative correlation with the Hopkins Verbal Learning Test in NPS-negative group (*r* = −0.304, *p* = 0.034). The right PHG in HC group (*r* = 0.250, *p* = 0.038), and the left ICC (*r* = 0.262, *p* = 0.030), the left paORB (*r* = 0.398, *p* = 0.001) and left frontal pole (*r* = 0.291, *p* = 0.015) in c-ICDs group showed a positive correlation with Letter Number Sequencing. The right frontal pole was negatively correlated with the MOCA (*r* = −0.250, *p* = 0.039) and Semantic Fluency Test (*r* = −0.283, *p* = 0.019) in c-ICDs group. There was no significant correlation between other brain regions and neuropsychological assessments (*p* > 0.05). There was no significant correlation between other brain regions and neuropsychological assessments (*p* > 0.05).

## 4 Discussion

In this study, we investigate the impact of ICDs on PD patients from normal cognition to MCI. Notably, in the four-year follow up, we observed that patients with PD who exhibited ICDs had notably longer survival times compared to those who were NPS-negative, while the conversion rates not significantly differed. And the survival time and conversion rates were also not significantly differed between the p-ICDs group and c-ICDs group. In the brain structural analysis, the p-ICDs group showed increased cortical thickness in the left ICC relative to the c-ICDs group, and both the p-ICDs group, and the c-ICDs group showed decreased cortical thickness of left ICC than NPS-negative group. And the p-ICDs group exhibited thinning in the right PHG than the NPS-negative group. The correlation analysis revealed that the right PHG showed a positive correlation with the Hopkins Verbal Learning Test in p-ICDs and HC group, but showed a negative correlation with the Hopkins Verbal Learning Test in NPS-negative group.

The longer survival time of PD with ICDs from MCI was observed, which indicating a potential protection effect of ICDs. Similar phenomenon were reported previously, Thomas et al. found that ICDs were associated with an increased psychiatric burden at baseline, but they were also linked to a better cognitive prognosis, it is believed that PD patients with ICDs at baseline, the intake of a certain dose of dopamine agonists (DA) leads to drug-induced overstimulation, impairing the top-down inhibitory control of ICDs, which somewhat delays the decline in cognitive function (37). Similarly, Chiara et al. found that PD patients with ICDs exhibited relatively lower cognitive decline over time (22, 38), which was then attributed to the drug-induced overstimulation exerted relatively preservation on prefrontal cognitive functions. Such possibility should also take in to consideration currently. In this work, patients were only receiving therapy of dopamine to control PD symptom. Our findings might result from the different reaction of the brain of PD patients with ICDs on the dopamine therapy, and further investigation were warranted. And with the not significantly differed conversion rates between patients with or without ICDs in 4 years, we speculate that the incidence of MCI in PD patient was certainly fixed, and dopamine induced effects in PD patients with ICDs might delayed the occurrence of MCI.

In our study, patients with ICDs were divided into p-ICDs group, PD with only ICDs, and c-ICDs group, PD with ICDs and other NPS. Relative to healthy controls, all three groups (p-ICDs, c-ICDs, and NPS) exhibited shared cortical alterations in the cingulate cortex, parahippocampal gyrus (PHG), and frontal regions, which correlate with deficits in emotional regulation, cognitive control, memory, and executive function (39–41). These structural changes are consistent with previous reports on PD (42, 43). Besides changes associated with PD, cortical thinning at left ICC was observed in p-ICDs group and c-ICDs group in relation to NPS negative group. The ICC is closely related to emotional process and regulation, and important in cognitive control and executive function (41, 44). The cortical atrophy of ICC was also reported in neurodegenerative diseases and psychiatric disorders, such as PD, AD and schizophrenia (45–48). Prior investigations have demonstrated that PD patients with ICDs exhibit greater cortical thickness in the left ICC following pharmacological induction compared to non-ICD counterparts, which was then assumed to be associated with drug effects and reward mechanisms (49). In our study, PD subjects did not receive dopamine treatment at baseline, and the drug effects and reward mechanisms maybe not activate. Notably, in the decreased cortical thickness of ICC in relation to NPS-negative group, the c-ICDs group showed even thinner ICC than p-ICD group. Given on our analysis was conducted at the baseline, and the dopamine therapy after image acquisition. Such phenomenon might result from two possibilities, the confounding effects of other NPS and/or the differed reaction to dopamine therapy, and further research is needed to validate these hypotheses. Additionally, PD participants from the PPMI cohort received standardized drug therapy during the follow-up period. Previous study reported the magnitude of polytherapy and anticholinergic drugs burden does not appear to modulate PD-MCI risk (50). in other words, the cognitive impact of impulse control disorders (ICDs) in Parkinson’s disease appears independent of medication classes or dosage levels.

Besides, the p-ICDs group exhibited thinning in the right PHG at baseline. Although previous study have shown that the PHG of PD patients with ICDs is slightly thicker than that of patients without ICDs after dopamine treatment, no significant statistical difference was observed, They believe that the structural changes are secondary to neuroplastic adaptations related to non-physiological dopaminergic stimulation (49). Our subjects had not received dopamine therapy at baseline, the thinning of the PHG at baseline may be due to the dopamine-induced reward mechanism not being activated. In addition, we found that the PHG in the c-ICDs group was slightly thinner than in PD patients without NPS, although this difference was not statistically significant, possibly due to the confounding effects of other NPS.

Additionally, we observed a positive correlation between the cortical thickness of right PHG and HVLT in p-ICDs and HC group, suggesting a potential link between ICDs and memory function in PD patients. Recent study also reported that PD with ICDs and normal elderly exhibited poor performance in language learning and memory functions (51, 52), notably, we found that the PHG thinning is accompanied by memory improvement in NPS-negative group, which might be that the brain maintains certain cognitive functions through adaptation and compensation mechanisms (53).

This study has several limitations. First, the distribution of other neuropsychiatric symptoms in this study is uneven, for example, the number of NPS-negative group is too small, although our results are interpretable, a larger and more evenly distributed sample is needed to further investigate the mediating effects of each psychiatric symptom on cognitive function. Second, our imaging structural study is limited to the baseline, and the imaging modalities are relatively single. Further longitudinal design and multimodal imaging analysis are needed to confirm our results.

In conclusion, by survival analysis and cortical structural analysis, this study provides valuable imaging markers and supplementary information on the impact of ICDs on cognitive decline in PD patients. It suggests that ICDs may play a protective role in cognitive ability, potentially delaying the onset of MCI in PDNC patients and being accompanied by corresponding changes in cortical thickness.

## Data Availability

The datasets presented in this study can be found in online repositories. The names of the repository/repositories and accession number(s) can be found at: https://www.ppmi-info.org/.

## Footnotes

^1^ http://www.ppmi-info.org

^2^V6.0.0, http://surfer.nmr.mgh.harvard.edu/.
