# Supplementary material for: The impact of impulse control disorders on cognitive decline in de novo Parkinson’s disease: a study based on structural MRI
Source: Front Neurol. 2025 Apr 25;16:1565046. doi: 10.3389/fneur.2025.1565046 (PMC12061859; doi:10.3389/fneur.2025.1565046)

**Table S1** The number of conversions and conversion rates for each group.

|  | p-ICDs(n=81) | c-ICDs(n=69) | NPS-negative(n=50) | *χ*2 | P value |
| --- | --- | --- | --- | --- | --- |
| MCI-convert  MCI-nonconvert | 16  65 | 19  50 | 19  31 | 5.34 | 0.073 |
| Conversion rate | 20% | 28% | 38% |  |  |

**Figure S1** Scatter plot of correlation results between certain significant brain regions and neuropsychological tests.


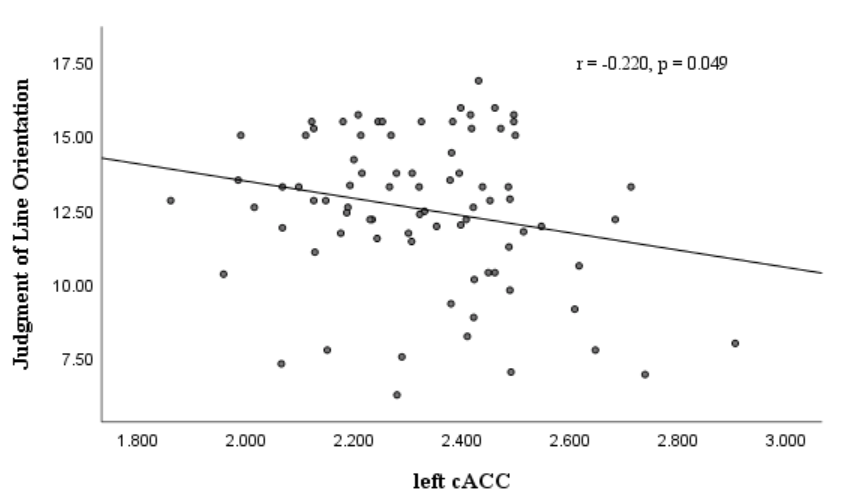

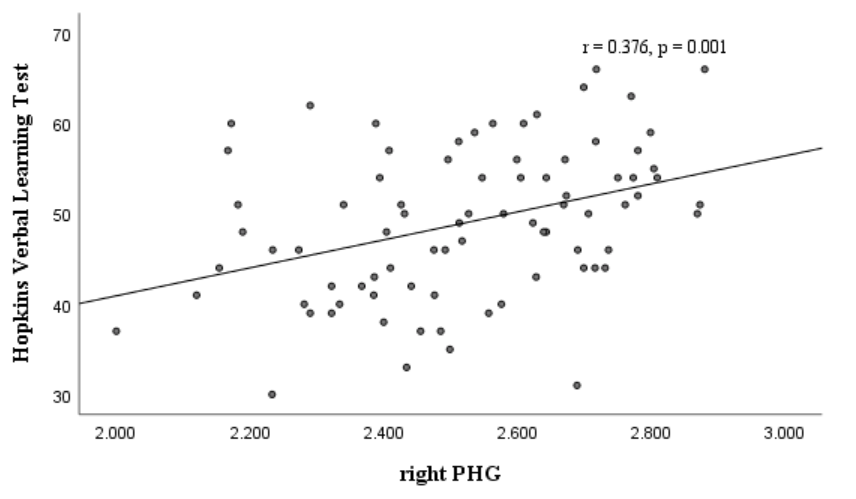

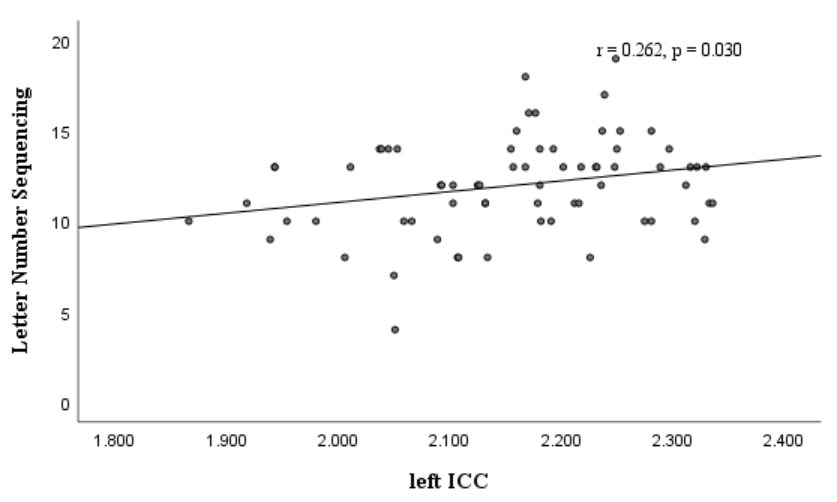

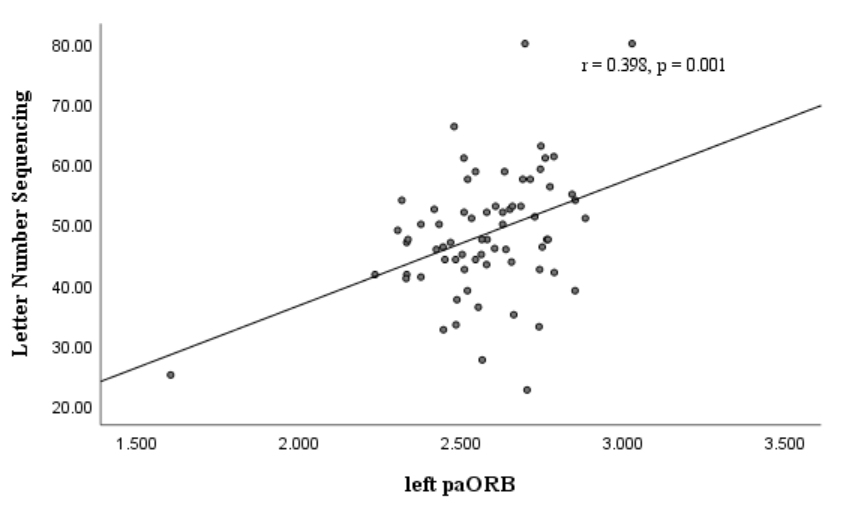

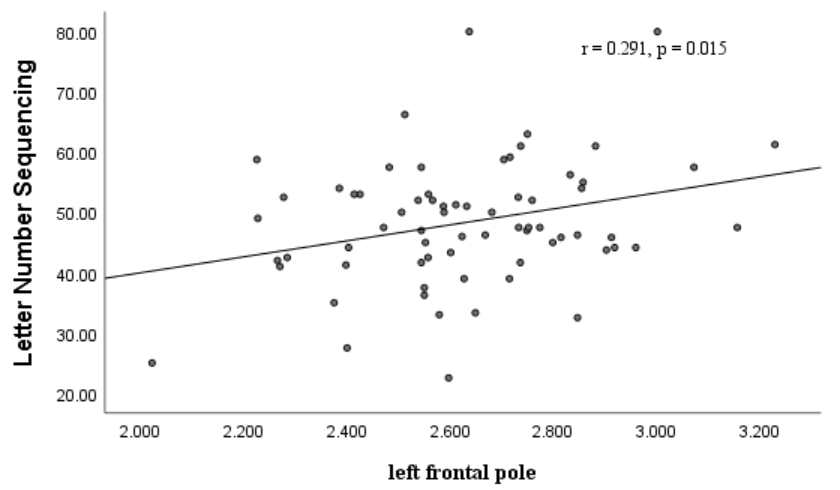

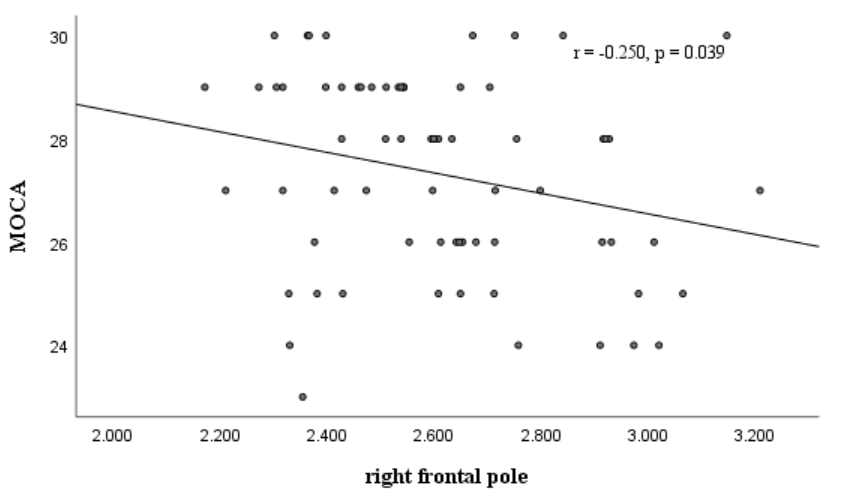

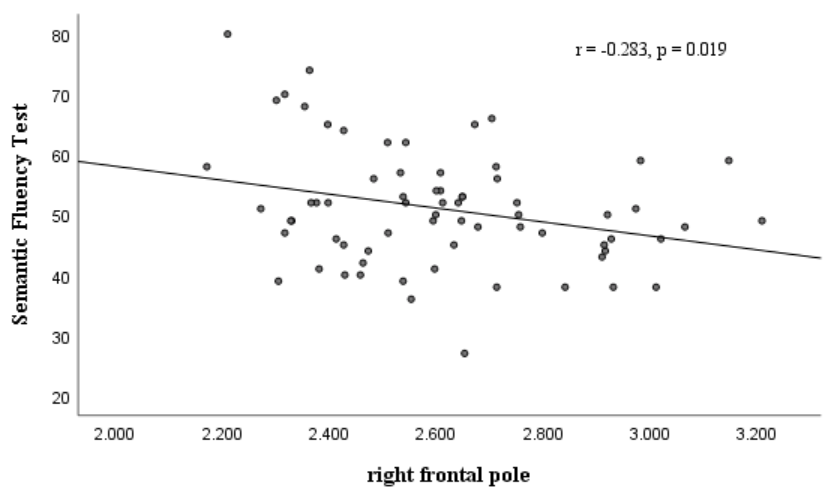

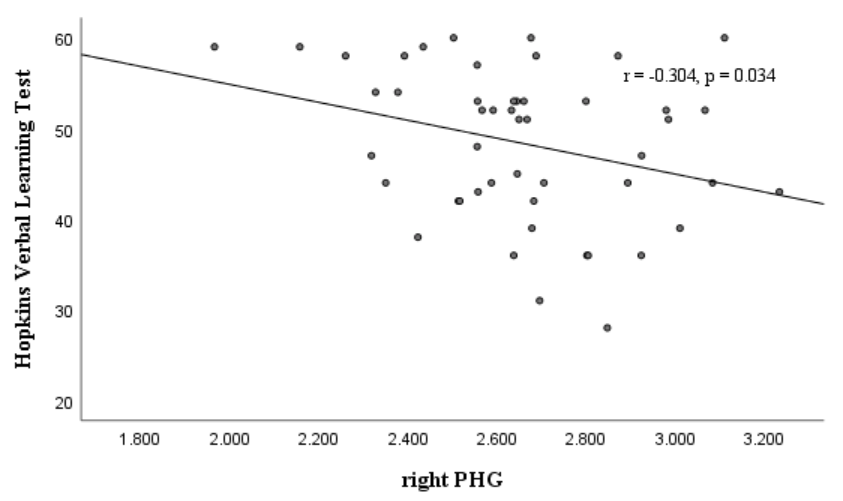

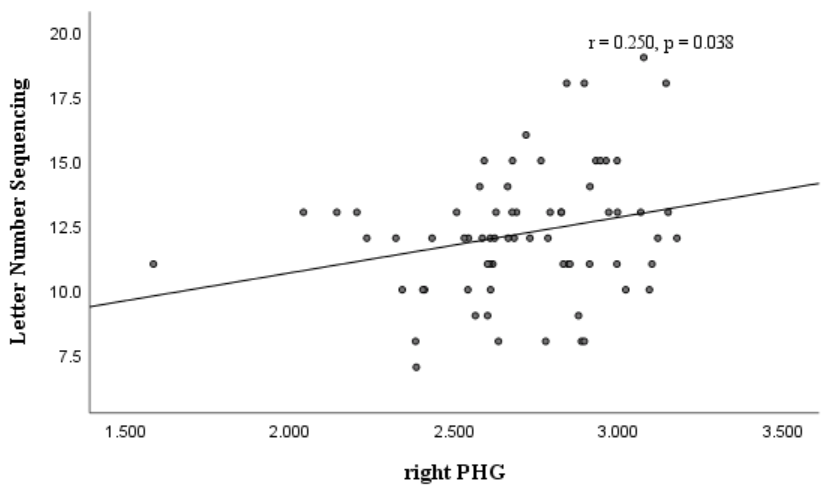


**FigureS2 The effect size curve among the p-ICDs, c-ICDs, NPS-negative group.**


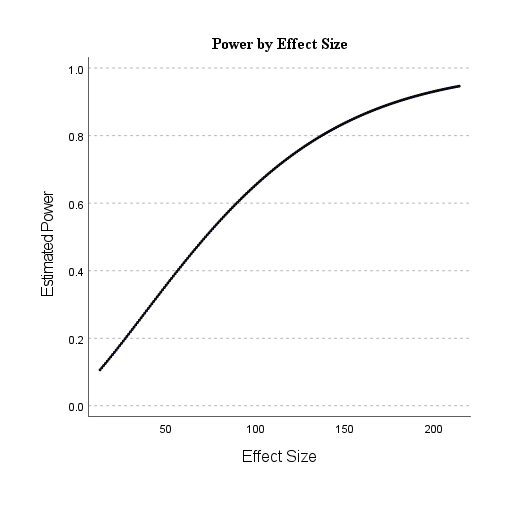

Supplement: Supplementary file 1 [file Supplementary_file_1.docx]
